# Prediction of microdissection testicular sperm extraction outcome in men with idiopathic nonobstruction azoospermia

**DOI:** 10.1097/MD.0000000000019934

**Published:** 2020-05-01

**Authors:** Han Zhang, Qi Xi, Xinyue Zhang, Hongguo Zhang, Yuting Jiang, Ruizhi Liu, Yang Yu

**Affiliations:** Center for Reproductive Medicine, Center for Prenatal Diagnosis, First Hospital, Jilin University, Changchun, China.

**Keywords:** hormone, microdissection testicular sperm extraction, non-obstructive azoospermia, sperm retrieval rate, testicular volume

## Abstract

The aim of the present study is to assess whether the preoperative clinical indicators have an impact on sperm retrieval rate (SRR) in men with idiopathic nonobstructive azoospermia (NOA).

We retrospectively studied 241 consecutive men with NOA who underwent microdissection testicular sperm extraction from 2016 to 2019 in the Reproductive Medicine Center, including 154 patients diagnosed with idiopathic NOA. They were grouped according to preoperative indicators, including average testicular volume, follicle-stimulating hormone (FSH), luteinizing hormone, Testosterone (T), and pathology, respectively.

The overall SRR was 20.0% (31/155). Men with testicular volume of ≤5 mL had significant higher SRR than men with testes 5 to 10 and ≥10 mL (35.6% vs 12.3%, *P* = .002; 35.6% vs 16.2, *P* = .049, respectively). The SRR in men with FSH ≥ 24.8 mIU/mL was significant higher, compared with FSH level of 12.4 to 24.8 mIU/mL (32.6% vs 15.8%, *P* = .033). Men with Sertoli cell-only had significantly lower SRR than other pathological type (8.1%). Men with an FSH ≥ 24.8 mIU/mL in testicular volume ≤5 mL group had a significantly higher SRR than FSH level of 12.4 to 24.8 mIU/mL in testicular volume of ≤5 to 10 mL group (44.0% vs 11.4%, *P* = .002). Men with a luteinizing hormone level of 8.6 to 17.2 mIU/mL in testicular volume of 5 to 10 mL group had a poor prognosis, with an SRR of only 6.5%.

Severely reduced testicular volume (≤5 mL) and severely increased FSH level (≥24.8 mIU/mL) had the better sperm retrieval outcome, which can be used as independent predictors in men with idiopathic NOA. And a combination of testicular volume and the hormone seemed to be useful in further increase predictive value.

## 1 Introduction

Nonobstructive azoospermia (NOA) due to testicular failure affects approximately 1% of all men and 10% to 15% of infertile men.^[1]^ Intracytoplasmic sperm injection combined with microdissection testicular sperm extraction (micro-TESE) is currently the first-line treatment option for men with NOA.^[2,3]^ Due to the different etiology of NOA, including congenital, acquired, and idiopathic etiology, sperm retrieval rate (SRR) varies greatly.^[4]^ In the congenital etiology, men with Klinefelter syndrome have an SRR of 40 to 50%.^[5–7]^ SRR in men with Y-chromosome microdeletions correlates with azoospermia factor (AZF) microdeletion sites: the likelihood of sperm retrieval in patients with complete AZFa and AZFb microdeletions was virtually zero and therefore TESE procedures were contraindicated.^[8,9]^ In the acquired etiology, SRR in men with orchitis and chemotherapy is related to the severity of testicular damage.^[10]^ However, the mechanism of idiopathic NOA is unclear and assumed to be caused by environmental pollution or genetic abnormalities, it is difficult to predict the SRR.

Various studies have been conducted on the predictors for presence of spermatozoa within the testis. Histological is the best predictor of a positive surgical sperm retrieval in patients with NOA.^[11]^ Follicle-stimulating hormone (FSH), luteinizing hormone (LH), testicular volume, and paternal age seemed to have poor predictive value for successful micro-TESE.^[12–14]^ However, age and testosterone can predict the success of sperm retrieval in patients with Klinefelter syndrome.^[5]^ Testicular size ≥ 15 mL and FSH 10 to 15 mIU/mL had the worst SRR in men with Sertoli cell-only syndrome (SCOS).^[15]^ The possible reason was that most studies had no further identification in etiology or pathology classification. Therefore, it is worth exploring the way to further identify SRR and reduce the unnecessary hazards. Especially for idiopathic NOA, it is necessary to classify clinical indicators due to unknown pathogenic mechanism.

Only a few studies evaluate the presence of spermatozoa in men with idiopathic NOA. Levels of anti-Mullerian hormone (AMH) and the ratio AMH-to-total testosterone achieved useful predictor for sperm retrieval.^[16]^ Tsujimiura et al showed the equation related to hormones should be useful in guiding doctors who recommend microdissection TESE for patients with NOA.^[17]^ To date, the prediction of micro-TESE outcome in men with idiopathic NOA are not well documented. Therefore, the aim of the present study is to assess whether the preoperative clinical indicators have an impact on SRR in men with idiopathic NOA.

## 2 Materials and methods

This was a retrospective observational study that received institutional review board approval from the Medical Ethics Committee of First Hospital of Jilin University and written informed consent was obtained from all patients.

### 2.1 Subjects

We retrospectively studied 241 consecutive men with NOA who underwent micro-TESE from 2016 to 2019 in the Reproductive Medicine Center of the First Clinical Hospital of Jilin University, including 154 patients diagnosed with idiopathic azoospermia. All patients were confirmed to have azoospermia by analyzing of at least 2 centrifuged semen samples according to World Health Organization criteria and underwent micro-TESE by the same surgeon. Preoperative factors, including age, the levels of FSH, LH and testosterone, any presence of varicocele, and any history of testicular cancer, orchitis, or cryptorchidism were analyzed. Testicular volume was measured by ultrasound. The average volume of the both testes was used for analyzed. If the patient had only 1 testis, it was used in the calculation. All men were subjected to karyotype and Y chromosome microdeletion analysis. Exclusion criteria: men with varicocele, testicular cancer, orchitis, cryptorchidism, abnormal karyotype, Y chromosome microdeletion, and other known etiology of NOA were excluded. All the procedures were performed by the same surgeon.

Patients with NOA were grouped according to preoperative indicators:

(1) They were classified into 3 groups based on an average testicular volume, namely ≤5 mL (severe small-volume), 5 to 10 mL (mild small-volume), and ≥10 mL (normal-volume).
(2) They were classified into 3 groups based on FSH and LH level, respectively, namely ≤12.4 and 8.6 mIU/mL (normal level), 12.4 to 24.8 and 8.6 to 17.2 mIU/mL (mild high level), ≥24.8 and ≥ 17.2 mIU/mL (severely high level).
(3) They were divided into 2 groups according to low (≤9.9 nmol/L) and normal (>9.9 nmol/L) testosterone.

### 2.2 Micro-TESE procedure

The procedure of micro-TESE has been described in our previous article.^[18]^ Briefly, under general anesthesia, make a midline scrotal incision and open the tunica vaginalis to expose the tunica albuginea. An equatorial incision was made over the tunica albuginea using a surgical microscope, taking care to avoid vasculature injury. Microdissection was then performed to identify larger and more opaque seminiferous tubules, which were considered more likely to contain sperm. The specimens were immediately examined by an embryologist in the operating room. If no sperm were observed intraoperatively, the testicular tissue was thoroughly examined for the presence of sperm by another embryologist in the embryology laboratory 12 to 24 hours later. Tissue specimen was placed in Bouin solution and sent for histopathological analysis.

### 2.3 Statistical analysis

All statistical data were analyzed with SPSS, version 22.0 (SPSS Inc., IBM, USA). For quantitative data such as testis volume, age, and hormone levels, independent-sample t test was performed to compare. The qualitative variables such as SRR was evaluated by the Chi-squared or Fisher exact test. *P* < .05 was considered statistically significant.

## 3 Result

### 3.1 Overall patient population

The mean age (range) of 155 idiopathic NOA patients was 31.1 (22–46) years in the present study. The overall SRR was 20.0% (31/155). Men with testicular volume of ≤5 mL had significantyl higher SRR than men with testes 5 to 10 mL and ≥10 mL (35.6% vs 12.3%, *P* = .002; 35.6% vs 16.2, *P* = .049, respectively) (Table 1). The SRR in men with FSH ≥ 24.8 mIU/mL was significantly higher, compared with FSH level of 12.4 to 24.8 mIU/mL (32.6% vs 15.8%, *P* = .033). There was no difference in SRR among LH groups and testosterone groups (Table 2). Men with Sertoli cell-only had a significantly lower SRR than other pathological type (Hypospermatogenesis: 8.1% vs 71.4%, *P* < .001; maturation arrest: 8.1% vs 36.4%, *P* < .001; tubular hyalinization: 8.1% vs 50%, *P* < .001, respectively) (Table 3).

**Table 1:**
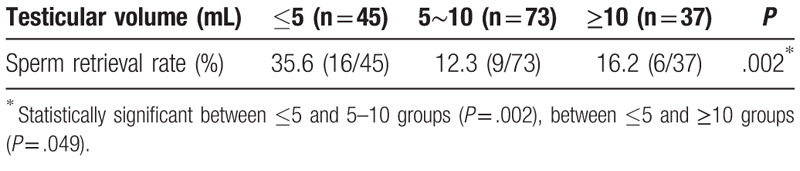
Sperm retrieval rate of testicular volume subgroups in the study group.

**Table 2:**
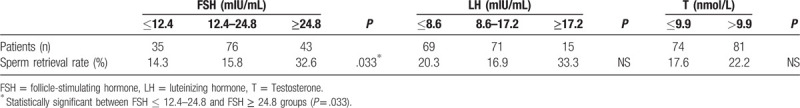
Sperm retrieval rate of hormone subgroups in the study group.

**Table 3:**
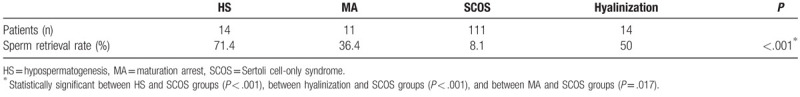
Sperm retrieval rate of pathological types in the study group.

### 3.2 Subpopulation analysis

**Figure 1:**
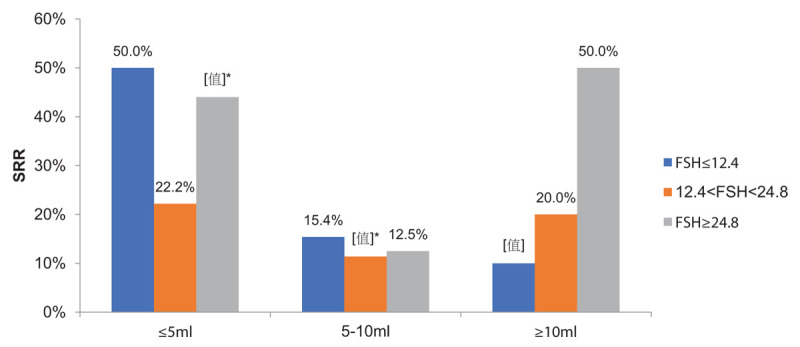
Sperm retrieval rate of testicular volume combined with follicle-stimulating hormone (FSH) subgroups. ^∗^Statistically significant between FSH ≥ 24.8 mIU/mL in testicular volume ≤5 mL group and FSH level of 12.4–24.8 mIU/mL in testicular volume of ≤5–10 mL group(*P* = .002).

**Figure 2:**
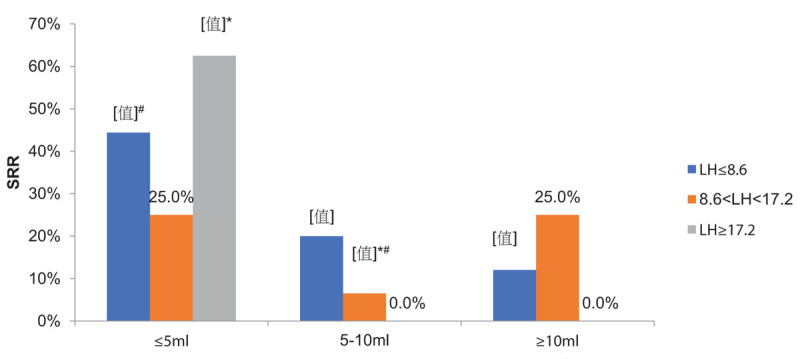
Sperm retrieval rate of testicular volume combined with luteinizing hormone (LH) subgroups. ^∗#^LH level of 8.6–17.2 mIU/mL in testicular volume of 5–10 mL group had statistically significantly lower than LH ≥ 17.2 mIU/mL (*P* = .001) or LH ≤ 8.6 mIU/mL in testicular volume of ≤ 5 mL group (*P* = .002).

SRRs stratified by hormone levels and testicular volumes are presented in Figures 1 and 2. Men with an FSH ≥ 24.8 mIU/mL in testicular volume ≤5 mL group had a significantly higher SRR than FSH level of 12.4 to 24.8 mIU/mL in testicular volume of ≤5 to 10 mL group (44.0% vs 11.4%, *P* = .002) (Fig. 1). Men with an LH level of 8.6 to 17.2 mIU/mL in testicular volume of 5 to 10 mL group had a poor prognosis, with an SRR of only 6.5%, which was significantly lower than the men with an LH ≥ 17.2 mIU/mL or LH ≤ 8.6 mIU/mL in testicular volume of ≤5 mL group (62.5% vs 6.5%, *P* = .001) (Fig. 2). Men with a testosterone > 9.9 nmol/L in testicular volume ≤5 mL group had a significantly higher SRR than both groups in testicular volume of ≤5 to 10 mL group (52.9% vs 12.5%, *P* = .019; 52.9% vs 12.2%, *P* = .003, respectively).

## 4 Discussion

Micro-TESE is an effective method for treating patients with NOA, but there is a lack of clinically accurate predictive indicators of sperm retrieval outcomes at testicular surgery. At present, it can be determined that the sperm cannot be obtained in patients with complete AZFa and AZFb microdeletions.^[8,9]^ The others can only confirm the presence of sperm in the testes after surgery. However, doctors can provide SRR consultation to NOA patients for different causes or pathologies to help them make better choices. Moreover, combining the etiology with other indicators will lead to better treatment procedure. For example, micro-TESE and frozen sperm should be taken after puberty for patients with Klinefelter syndrome due to the declining SRR with increasing age.^[5]^ Testicular volume and age were identified as independent predictors of sperm retrieval for men with a history of cryptorchidism.^[19]^ But the SSR of idiopathic azoospermia varied widely and was difficult to predict.

The present study was to further classify the preoperative clinical indicators to predict SRR in patients with idiopathic NOA. Our data showed that patients with severely small testicular volume (≤5 mL) and severely increased FSH level (≥24.8 mIU/mL) had the best sperm retrieval outcome. This was different from previous reports. Bryson et al showed severe testicular atrophy did not affect the success of microdissection testicular sperm extraction.^[13]^ Ramasamy et al showed high serum FSH levels did not affect SRR. Most studies found no difference in testicular volume and FSH between the successful sperm retrieval group and the no sperm retrieval group in men with NOA.^[20,21]^ The possible reasons involved 2 aspects: First, most studies had no further identification in etiology classification, such as Klinefelter syndrome was the most common in men with severe small testicles. Second, our study also showed SCOS had significant lower SRR than other pathological type. Anniballo et al found men with pure SCOS had the normal diameter tubules and normal testicular size, which was difficult to find spermatozoa.^[22]^ Therefore, the pathological classification of large testes and normal FSH was more likely to be SCOS, and the SRR was reduced.

Moreover, we compared the SRR by combining the testicular volume subgroup with the hormone subgroup. We found an interesting result that patients with a slight decrease testicular volume combined with a slight increase in FSH/LH had poor SRR. However, patients with severely reduced testicular volume combined with severely increased FSH/LH had high SRR. This was different from our conventional perception, because severely small testes and high FSH represented spermatogenesis failure. Most studies have shown that testicular volume and hormone level did not affect fertility.^[12,13]^ However, studies on patients with idiopathic NOA have shown that severely reduced testicular volume and severely higher FSH/LH had better sperm retrieval rate. The possible reason was that the slightly increased indicators may be related to unknown congenital genetic causes or complete spermatogenic arrest, which had extremely low SRR. These causes were less destructive to the overall testicular tissue structure, so these indicators had not changed much. A slight decrease testicular and normal/slightly lower testosterone also met the above conditions.

In conclusion, in addition to pathology, severely reduced testicular volume (≤5 mL) and severely increased FSH level (≥24.8 mIU/mL) had the better sperm retrieval outcome, which can be used as independent predictors in men with idiopathic NOA. And a combination of testicular volume and the hormone seemed to be useful in further increase predictive value.

## Author contributions

**Conceptualization:** Qi Xi, Hongguo Zhang.

**Data curation:** Qi Xi, Yuting Jiang.

**Formal analysis:** Xinyue Zhang.

**Funding acquisition:** Ruizhi Liu.

**Investigation:** Han Zhang, Hongguo Zhang.

**Project administration:** Ruizhi Liu.

**Resources:** Hongguo Zhang.

**Writing – original draft:** Han Zhang.

**Writing – review & editing:** Xinyue Zhang.
